# Human Bone Proteomes before and after Decomposition: Investigating the Effects of Biological Variation and Taphonomic Alteration on Bone Protein Profiles and the Implications for Forensic Proteomics

**DOI:** 10.1021/acs.jproteome.0c00992

**Published:** 2021-03-08

**Authors:** Hayley L. Mickleburgh, Edward C. Schwalbe, Andrea Bonicelli, Haruka Mizukami, Federica Sellitto, Sefora Starace, Daniel J. Wescott, David O. Carter, Noemi Procopio

**Affiliations:** † Department of Cultural Sciences, Linnaeus University, Kalmar 352 52, Sweden; ‡ Forensic Anthropology Center, Texas State University, San Marcos 78666, Texas, United States; § Forensic Science Research Group, Faculty of Health and Life Sciences, Northumbria University, Ellison Building, Northumbria University Newcastle, Newcastle Upon Tyne NE1 8ST, U. K.; ∥ Dipartimento di Chimica, University of Turin, Via P. Giuria 7, 10125 Turin, Italy; ⊥ Forensic Sciences Unit, School of Natural Sciences and Mathematics, Chaminade University of Honolulu, Honolulu 96816, Hawaii, United States

**Keywords:** forensic proteomics, forensic taphonomy, bone mineral density, post-mortem interval estimation, age-at-death estimation, human decomposition, forensic microbiology

## Abstract

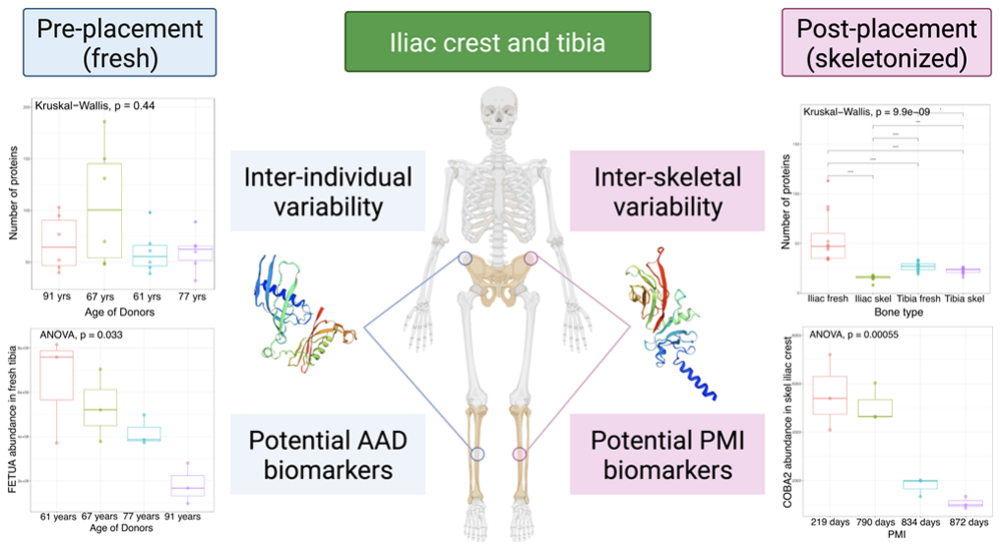

Bone proteomic studies using animal proxies and skeletonized human remains have delivered encouraging results in the search for potential biomarkers for precise and accurate post-mortem interval (PMI) and the age-at-death (AAD) estimation in medico-legal investigations. The development of forensic proteomics for PMI and AAD estimation is in critical need of research on human remains throughout decomposition, as currently the effects of both inter-individual biological differences and taphonomic alteration on the survival of human bone protein profiles are unclear. This study investigated the human bone proteome in four human body donors studied throughout decomposition outdoors. The effects of ageing phenomena (*in vivo* and post-mortem) and intrinsic and extrinsic variables on the variety and abundancy of the bone proteome were assessed. Results indicate that taphonomic and biological variables play a significant role in the survival of proteins in bone. Our findings suggest that inter-individual and inter-skeletal differences in bone mineral density (BMD) are important variables affecting the survival of proteins. Specific proteins survive better within the mineral matrix due to their mineral-binding properties. The mineral matrix likely also protects these proteins by restricting the movement of decomposer microbes. New potential biomarkers for PMI estimation and AAD estimation were identified. Future development of forensic bone proteomics should include standard measurement of BMD and target a combination of different biomarkers.

## 1 Introduction

Estimations of the time elapsed since death (post-mortem interval, PMI) and the age-at-death (AAD) are crucial in the forensic investigation of unidentified human remains. This information is important to distinguish between historical remains (>100 years old) and remains of medico-legal relevance (≤100 years old)^1,2^ and to narrow the search of missing persons for identification purposes.^3,4^ High precision, accuracy, and objectivity of PMI and AAD estimation methods are essential in order to be considered admissible in a court of law.

PMI estimation often relies on visual assessment of gross morphological changes of the body during decomposition,^5−7^ even though the rate of these changes is known to be highly variable.^8,9^ Accuracy of the PMI estimation decreases as decomposition progresses, and interobserver reliability differs depending on the method and the experience of the researcher.^9,10^ Biochemical techniques have shown promising results in the search for a precise and accurate method to estimate late PMI in human bone; however, these methods are yet to be validated for use in forensic contexts.^11−14^

Standard AAD estimation methods are based on the examination of the morphological characteristics of the remains^15^ and require the evaluation of several different skeletal elements.^16^ Different methods are applied to juveniles and adults.^17,18^ Limitations of these methods include a high interobserver variability,^15^ inter- and intra-population variability with increasing AAD,^19^ lack of consensus regarding the evaluation of the errors,^20^ poor precision in adult aging in comparison with juvenile and adolescent aging,^4^ and the requirement for remains to be as complete as possible.^20^

In recent years, bone proteomic methods have been demonstrated to be highly promising for the development of precise, accurate, and objective PMI and AAD estimation methods and require only small samples of bone. Proteins are thought to be relatively stable in bone and have been successfully extracted from archaeological^21−25^ and paleontological specimens,^26−29^ making them a promising target for forensic applications.^30^ Studies conducted using animal models (*e.g.*, *Sus scrofa* and *Bos bovid*) focused on inter- and intra-individual comparisons and monitored changes in the bone proteomes associated with progressing decomposition stages. These studies revealed inter- and intra-skeletal proteomic variability^31^ and identified potential biomarkers for AAD^31^ and PMI estimations.^32^ In addition, the burial environment was found to affect the proteome recovered from archaeological specimens.^33^ However, the development of bone proteomic methods for forensic science remains impeded by the fact that it is unknown how representative animal models are for human specimens. Moreover, it is largely unknown how taphonomic processes and inter-individual variation (both *in vivo* and at the time of death), including underlying health conditions, affect the survival and extraction of bone protein profiles in humans.

A recent study conducted on human bones, collected from a cemetery in Southeast Spain, provided promising new insights on the estimation of broad PMI ranges (5–20 years) in humans using protein biomarkers in proximal femoral bone.^34^ The study identified 32 proteins which could be used in conjunction to discriminate between PMIs greater or smaller than 12 years.^34^ The sampled individuals were subjected to similar taphonomic conditions, and PMIs were greater than 7 years in all but one case. While the study was conducted on a relatively large sample (*n* = 40), inter-individual and inter-skeletal comparison of bone protein profiles at different stages of decomposition of the body was not possible as only one skeletal element was available per individual, and bones were sampled only after decomposition of the soft tissues. For the further development, and ultimately validation, of forensic proteomics to estimate PMI, the study of changes in human bone protein profiles from the fresh stage of decomposition to the skeletonized stage is crucial.

In this study, we aimed to investigate the effects of taphonomy and biological variation on the recovery and variability of the human bone proteome and evaluate potential avenues to develop a broadly applicable, standardized method of PMI and AAD estimation in human remains in advanced state of decomposition.^35^ The proteomes of anterior midshaft tibia and iliac crest samples from four body donors of known AAD (two buried and two placed in an open pit), taken shortly after death and upon complete skeletonization of the body, were analyzed to investigate (1) whether the previously identified potential biomarkers for PMI and AAD are applicable to human bones with lower PMIs, (2) whether additional potential biomarkers for PMI/AAD estimation could be identified, (3) whether the human bone proteome is subject to inter-skeletal (among different skeletal elements of the same individual), intra-skeletal (within the same skeletal element), and inter-individual (within the same skeletal element among different individuals) variability, and (4) the role decomposition, depositional environment and taphonomy, and season play in bone proteome survival.

## 2 Experimental Section

### 2.1 Body Donations

The body donations of four females aged between 61 and 91 years old were placed unclothed to decompose at the Forensic Anthropology Research Facility (FARF), the outdoor human decomposition facility associated with the Forensic Anthropology Center at Texas State University (FACTS), between April 2015 and March 2018. While the targeted bone proteins in this study are not thought to differ between males and females, only post-menopausal female individuals were included, in order to exclude biological sex and major hormonal differences as a potential variable from the study. Two body donations (D2 and D3) were buried with soil in shallow hand dug pits. Two body donations (D1 and D4) were placed in pits of similar dimensions, which remained open throughout the experiment. Open pits were covered with metal cages to protect the remains from large scavengers. The sample size in this study reflects general trends in human decomposition research, in which larger samples—like those used in clinical studies—can be difficult to obtain for practical, logistical, and ethical reasons. While animal analogues such as pigs can be used to alleviate some limitations associated with small sample sizes, the study of human cadavers is important due to biological differences between humans and pigs, including anatomical differences in the digestive vasculature and molecular differences in adipose tissue.^36^

Data on body decomposition and weather were collected throughout the experiment and can be found in Supporting Information (Table 1). Additional information on FARF’s environment can be found in the Supporting Information. Gross decomposition was quantified using the total body score method following a study by Megyesi et al.^37^ Accumulated degree-days (ADD) were calculated using temperature data recorded on the facility (Supporting Information, Table 2).

### 2.2 Bone Sample Collection

Bone samples (*ca.* 1 cm^3^) of the anterior midshaft tibia and iliac crest (left) were collected prior to placement of the fresh body outside and upon retrieval of the completely skeletonized remains (right). The midshaft tibia was analyzed in this study because previous research has shown its great intra-skeletal and inter-individual proteomic reproducibility.^31^ The iliac crest was additionally targeted because this bone has naturally higher porosity and perfusion (*i.e.*, the circulation of blood through the tissue) compared to the long bones and because a higher amount of bacterial infiltration (post-mortem colonization by gut bacteria) is to be expected in iliac bone due to its proximity to the intestinal area. Proteomic comparison between two areas of the skeleton which naturally differ in bone density, perfusion of the bone, as well as proximity to large bacterial communities known to play a significant role in early and advanced decomposition of the body, can offer valuable insights into the role that both taphonomic and biological variables play in the survival of the bone proteome throughout decay. The total of 16 bone samples were stored in sterile plastic bags and immediately transferred to a lockable freezer at −80 °C. Samples were shipped overnight on dry ice to the Forensic Science Unit at Northumbria University, U.K. Upon arrival, the samples were immediately transferred to a lockable freezer at −18 °C, adhering to the U.K. Human Tissue Act under the license number 12495. The experiment was reviewed and approved by the ethics committee at Northumbria University, with the reference code 11623. All biological and bone sample data are provided in Table 1. Observations on bone condition (density and color) during sampling can be found in the Supporting Information.

**Table 1:** Biological and Bone Sample Data

donor	age at death (years)	sex	sample ID	lab ID (three extractions)	depositional context	placement date (dd-mm-yy)	collection date (dd-mm-yy)	*T* in days^a^
1	91	F	B1A-2-iliac	NP1-2-3	open pit	28-04-2015	28-04-2015	–1
1	91	F	B1A-2-tibia	NP4-5-6	open pit		28-04-2015	–1
1	91	F	B1C-2-iliac	NP7-8-9	open pit		03-12-2015	219
1	91	F	B1C-2-tibia	NP10-11-12	open pit		03-12-2015	219
2	67	F	B2A-2-iliac	NP13-14-15	burial	07-05-2015	07-05-2015	0
2	67	F	B2A-2-tibia	NP16-17-18	burial		07-05-2015	0
2	67	F	B2C-2-iliac	NP19-21-21	burial		17-08-2017	834
2	67	F	B2C-2-tibia	NP22-23-24	burial		17-08-2017	834
3	61	F	B3A-2-iliac	NP25-26-27	burial	24-06-2015	24-06-2015	0
3	61	F	B3A-2-tibia	NP28-29-30	burial		24-06-2015	0
3	61	F	B3C-2-iliac	NP31-32-33	burial		21-08-2017	790
3	61	F	B3C-2-tibia	NP34-35-36	burial		21-08-2017	790
4	77	F	B4A-2-iliac	NP37-38-39	open pit	19-10-2015	19-10-2015	–1
4	77	F	B4A-2-tibia	NP40-41-42	open pit		19-10-2015	–1
4	77	F	B4C-2-iliac	NP43-44-45	open pit		09-03-2018	872
4	77	F	B4C-2-tibia	NP46-47-48	open pit		09-03-2018	872

a T_0_ = day of burial/placement.

### 2.3 Sub-sampling and Sample Preparation

The 16 samples were defrosted prior to their analysis, then cleaned in deionized water for 3 h at room temperature, exchanging the water three times, once every hour. They were then dried in a fume cupboard at room temperature until completely dry. Bone samples were then secured in a table clamp for the sampling. Contamination between samples was prevented by using a double layer of aluminum foil within the clamp (in contact with the bone) and by using new foil double layers for each piece of bone sampled. The clamp was also cleaned in between each sampling step using 50% sodium hypochlorite (Sigma-Aldrich, U.K.), to further prevent contamination issues. Once the bone was secured in the clamp, Dentist’s Protaper Universal Hand Files (Henry Schein Minerva Dental, U.K.) were used to hand-drill ∼25 mg of fine bone powder three times (*i.e.*, three samplings were performed on the same bone fragment), in order to obtain three replicates for each of the bones analyzed. By sampling in different locations close together on the same bone, we obtained multiple biological samples. Since it is known that bone proteins can vary throughout the human skeleton and within individual bones, these biological replicates, in contrast to technical replicates, allow us to assess the degree of intra-bone variability and to establish whether inter-individual differences are greater than the intra-bone variability, as indicated in a previous study using pigs as proxies.^31^ Protaper files were changed between each sample, to prevent contamination. When the bone samples were too porous to obtain a fine bone powder (*e.g.*, iliac crest samples), small bone fragments were cut using the Protaper files, and ∼25 mg of bone fragments was collected for each of the three subsamples in order to have three replicates.

### 2.4 Micro Computed Tomography

During sampling of the bone, one of the specimens was observed to be considerably denser compared to the others. This difference was investigated non-invasively using micro computed tomography (μCT), in order to quantify the difference and to examine any potential relationship with the proteomic results. Microarchitectural and compositional properties were examined by means of μCT. Bone specimens were scanned with a NSI X5000 micro-CT system (North Star Imaging, Aliso Viejo, California, USA) operated at 79 kV and 0.185 mA. Voxel size was 117.095 μm for Tibia and 51.07 μm for the Crista Iliaca. BoneJ was employed to quantify certain material volume (BV) and total volume (TV) for the region of interest represented by the cortical area above the sampling area. A QRM-Micro-CT-HA D20 (QRM GmbH, Moehrendorf, Germany) calibration phantom was scanned under the same conditions. The mean gray scale values obtained from the attenuation histogram were used to fit the calibration curve of volumetric tissue mineral density (vTMD) gray scale values. These were employed to calculate vTMD values for the cortical area under analysis. Furthermore, volumetric bone mineral density values (vBMD) was calculated according to the following formula:
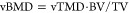


### 2.5 Protein Extraction

Overall, 48 samples were obtained from the 16 bone pieces and subjected to bone protein extraction following the protocol of Procopio and Buckley.^38^ Briefly, each sample was decalcified with 1 mL of 10 v/v % formic acid (Fisher Scientific, U.K.) for 6 h at 4 °C. After removing all the acid soluble fraction, the acid insoluble fraction was incubated for 18 h at 4 °C with 500 μL of 6 M guanidine hydrochloride/100 mM TRIS buffer (pH 7.4, Sigma-Aldrich, U.K.). The buffer was exchanged into 100 μL of 50 mM ammonium acetate (Scientific Laboratory Supplies, U.K.) with 10K molecular-weight cut off filters (Vivaspin 500 polyethersulfone, 10 kDa, Sartorius, Germany), and samples were then reduced with 4.2 μL of 5 mM dithiothreitol (DTT) (Fluorochem, U.K.) for 40 min at room temperature and alkylated with 16.8 μL of 15 mM iodoacetamide (Sigma-Aldrich, U.K.) for 45 min in the dark at room temperature. Samples were then quenched with another 4.2 μL of 5 mM DTT, then digested with 0.4 μg of trypsin (Promega, U.K.) for 5 h at 37 °C, and finally frozen. By adding 15 μL of 1 v/v % trifluoroacetic acid (TFA) (Fluorochem, U.K.), the digestion was stopped and the samples were then desalted, concentrated, and purified using OMIX C18 pipette tips (Agilent Technologies, U.S.A.) with 0.1 v/v % TFA as washing solution and 50 v/v % acetonitrile (ACN) (Thermo Fisher Scientific, U.K.)/0.1 v/v % TFA as a conditioning solution. Pipette tips were prepared with two volumes of 100 μL of 0.1 v/v % TFA and washed twice with 100 μL of 50 v/v % ACN/0.1 v/v % TFA. The sample was then aspirated into the tip at least ten times to efficiently bind peptides to the absorbent membrane. Finally, two washing steps with 100 μL of 0.1 v/v % TFA were performed, prior to peptides elution into 100 μL of 50 v/v % ACN/0.1 v/v % TFA. Purified peptides were left in the fume cupboard at room temperature with lids open to dry prior to their submission for LC–MS/MS analysis.

### 2.6 LC/MS–MS Analysis

Samples resuspended in 5 v/v % ACN/0.1 v/v % TFA were analyzed by LC–MS/MS using an Ultimate 3000 Rapid Separation LC (RSLC) nano LC system (Dionex Corporation, Sunnyvale, CA, USA) coupled to a Q Exactive Plus Hybrid Quadrupole-Orbitrap Mass Spectrometer (Thermo Fisher Scientific, Waltham, MA, U.S.A.). Peptides were separated on an EASY-Spray reverse phase LC Column (500 mm × 75 μm diameter (i.d.), 2 μm, Thermo Fisher Scientific, Waltham, MA, USA) using a gradient from 96 v/v % A (0.1 v/v % FA in 5 v/v % ACN) and 4 v/v % B (0.1 v/v % FA in 95 v/v % ACN) to 8 v/v %, 30 v/v %, and 50% B at 14, 50, and 60 min, respectively, at a flow rate of 300 nL min^–1^. Acclaim PepMap 100 C18 LC Column (5 mm × 0.3 mm i.d., 5 μm, 100 Å, Thermo Fisher Scientific) was used as trap column at a flow rate of 25 μL min^–1^ maintained at 45 °C. The LC separation was followed by a cleaning cycle with an additional 15 min of column equilibration time. Then, peptide ions were analyzed in the full scan MS scanning mode at 35,000 MS resolution with an automatic gain control (AGC) of 1 × 10^6^, injection time of 200 ms, and scan range of 375–1400 *m*/*z*. The top ten most abundant ions were selected for data-dependent MS/MS analysis with a normalized collision energy level of 30 performed at 17,500 MS resolution with an AGC of 1 × 10^5^ and maximum injection time of 100 ms. The isolation window was set to 2.0 *m*/*z*, with an underfilled ratio of 0.4%, dynamic exclusion was employed; thus, one repeat scan (*i.e.*, two MS/MS scans in total) was acquired in a 45 s repeat duration with the precursor being excluded for the subsequent 45 s.

### 2.7 Data Analysis and Statistical Analysis

Peptide mass spectra were then searched against the SwissProt_2019_11 database (selected for *Homo sapiens*, unknown version, 20,368 entries) using the Mascot search engine (version 2.5.1; www.matrixscience.com) for matches to primary protein sequences. This search included the fixed carbamidomethyl modification of cysteine as it results from addition of DTT to proteins. Deamidation (asparagine and glutamine) and oxidation (lysine, methionine, and proline) were considered as variable modifications. The enzyme was set to trypsin with a maximum of two missed cleavages allowed. Mass tolerances for precursor and fragmented ions were set at 5 ppm and 0.5 Da, respectively. It was assumed that all spectra hold either 2+ or 3+ charged precursors. Scaffold (version Scaffold_4.10.0, Proteome Software Inc., Portland, OR) was used to validate MS/MS-based peptide and protein identifications. Peptide identifications were accepted if they could be established at greater than 95.0% probability to maximize the reliability of the identifications. Peptide Probabilities from Mascot were assigned by the Scaffold Local FDR algorithm and by the Peptide Prophet algorithm^39^ with Scaffold delta-mass correction. Protein identifications were accepted if they could be established at greater than 90.0% probability and contained at least two identified peptides, in order to filter for the most accurate matches. This resulted in having a calculated decoy False Discovery Rate (FRD) of 0.06% for peptides and 1.9% for proteins. Protein probabilities were assigned by the Protein Prophet algorithm.^40^ Proteins that contained similar peptides and could not be differentiated based on MS/MS analysis alone were grouped to satisfy the principles of parsimony. Proteins sharing significant peptide evidence were grouped into clusters. Progenesis Qi for Proteomics (version 4.1; Nonlinear Dynamics, Newcastle, U.K.) was used to perform relative quantitation calculations using the recorded ion intensities (area under the curve) and averaging the N most abundant peptides for each protein (Hi–N method, where *N* = 3) and protein and post-translational modification identifications. In order to increase the reliability of the matches, peptide ions with a score of <28, which indicates identity or extensive homology (*p* < 0.05), were excluded from the analysis based on the Mascot evaluation of the peptide score distribution for the searched .mgf file originating from Progenesis (combining all the samples in a single experiment). To further improve the reliability of the findings, we implemented an additional level of filtering, excluding proteins with a peptide count of <2. Samples were grouped together using the between-subject design scheme in Progenesis, in order to compare selected groups of samples (*e.g.*, skeletonized *vs* fresh bones) and to calculate ANOVA *p*-values and maximum fold changes accordingly. The use of three extractions per targeted bone sample provided a sufficiently large data set for comparative analysis. To identify proteins of interest, proteins were selected that had an ANOVA *p*-value ≤ 0.05 and a maximum fold change ≥2. Common contaminants such as keratins were excluded from the interpretation of the results. Plots were carried out using R version 3.6.2 with packages dplyr, ggplot2, ggpubr, and patchwork packages. When plotting boxplots, for data following a normal distribution student’s *t*-test and one-way ANOVA and post-hoc pairwise comparisons were used to test mean differences, otherwise the Wilcoxon rank sum test and Kruskal Wallis test with post-hoc pairwise comparisons were used. STRING software version 11.0 was used to visualize functional links between the extracted proteins.^41^ The confidence score required for showing interactions was set to “high = 0.700.” The MCL clustering method was used to identify the clusters, with inflation parameter = 1.5.

## 3 Results

### 3.1 Proteomic Data

**Figure 1:**
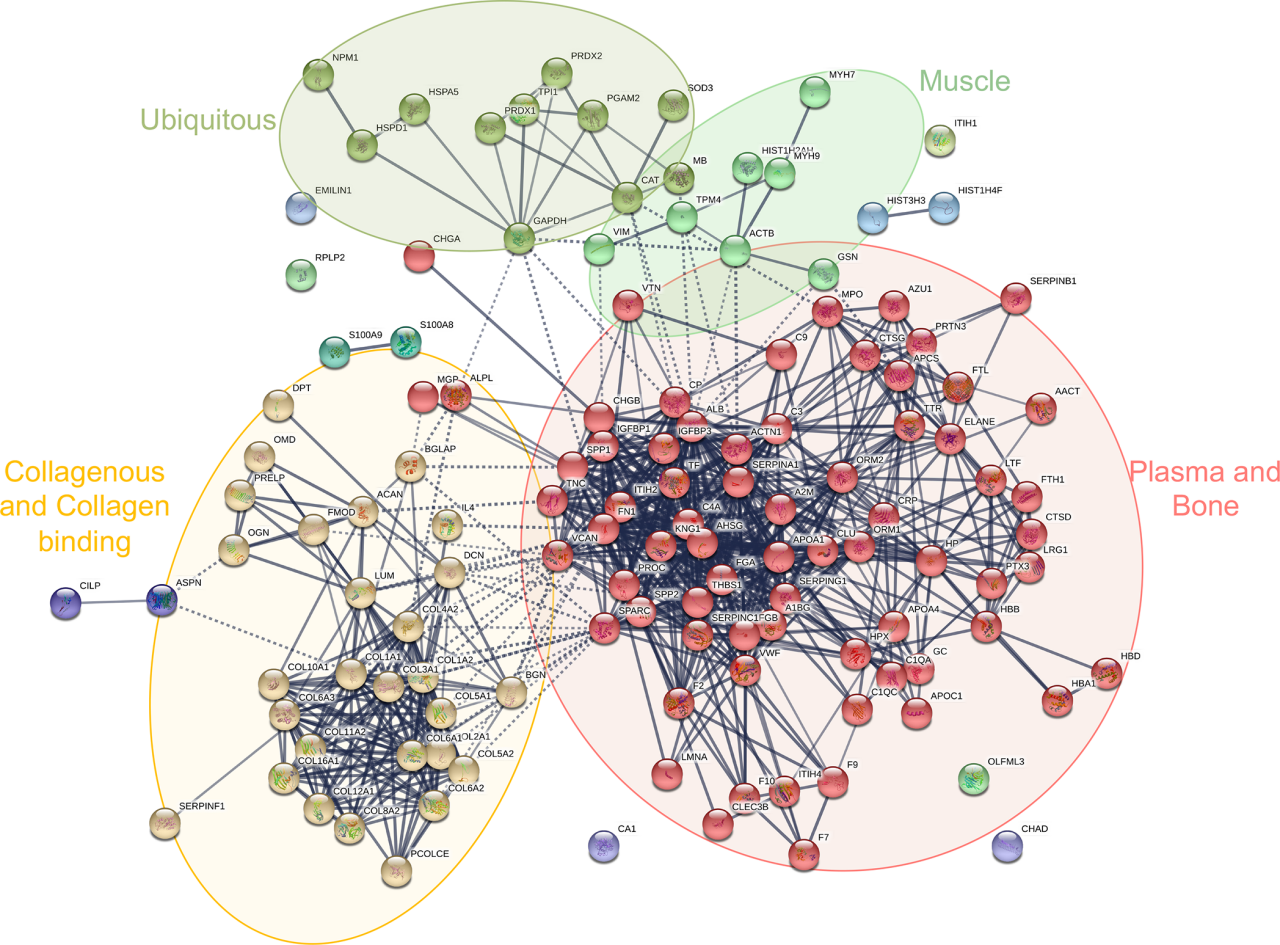
STRING protein network of the quantifiable proteins extracted from all samples. Immunoglobulin proteins (gene names IGHA1, IGHG2, IGHG3, IGKC, and IGLC2) were not found with STRING and are not represented in the figure. The light orange ellipse represents collagenous and collagen-binding proteins, the red one represents plasma and bone-related proteins, the yellow-green one at the top on the left side represents ubiquitous proteins, and the light green one at the top on the right side represents some muscle proteins. Other smaller clusters represent other types of proteins interacting less with the major clusters identified.

The proteome of both the midshaft tibia and the iliac crest of four human body donors sampled at “fresh” (PMI = 2–10 days) and at “skeletonized” (*i.e.*, when bodies did not have any adhering/desiccated soft tissue) stages of decomposition (PMI variable, between ∼5200 and ∼17,800 ADD, depending on the season of placement, see Supporting Information, Table 2) was analyzed. Three replicate extractions were taken from each bone, totaling 48 proteomic analyses (Supporting Information, Table 2). After refining the Progenesis results based on the number of unique peptides and on the ion score (see the Methods section), 133 quantifiable proteins including bone, plasma, ubiquitous, muscle, and extracellular matrix proteins were identified (Supporting Information, Data 1). The protein interaction network (Figure 1) showed a significant enrichment of interactions (PPI enrichment *p* < 1.0 × 10^–16^) and functional enrichments of specific GO terms for biological processes, cellular components, and molecular functions (Supporting Information, Data 2).

### 3.2 Human Proteomic Inter-Skeletal and Inter-Individual Variability

**Figure 2:**
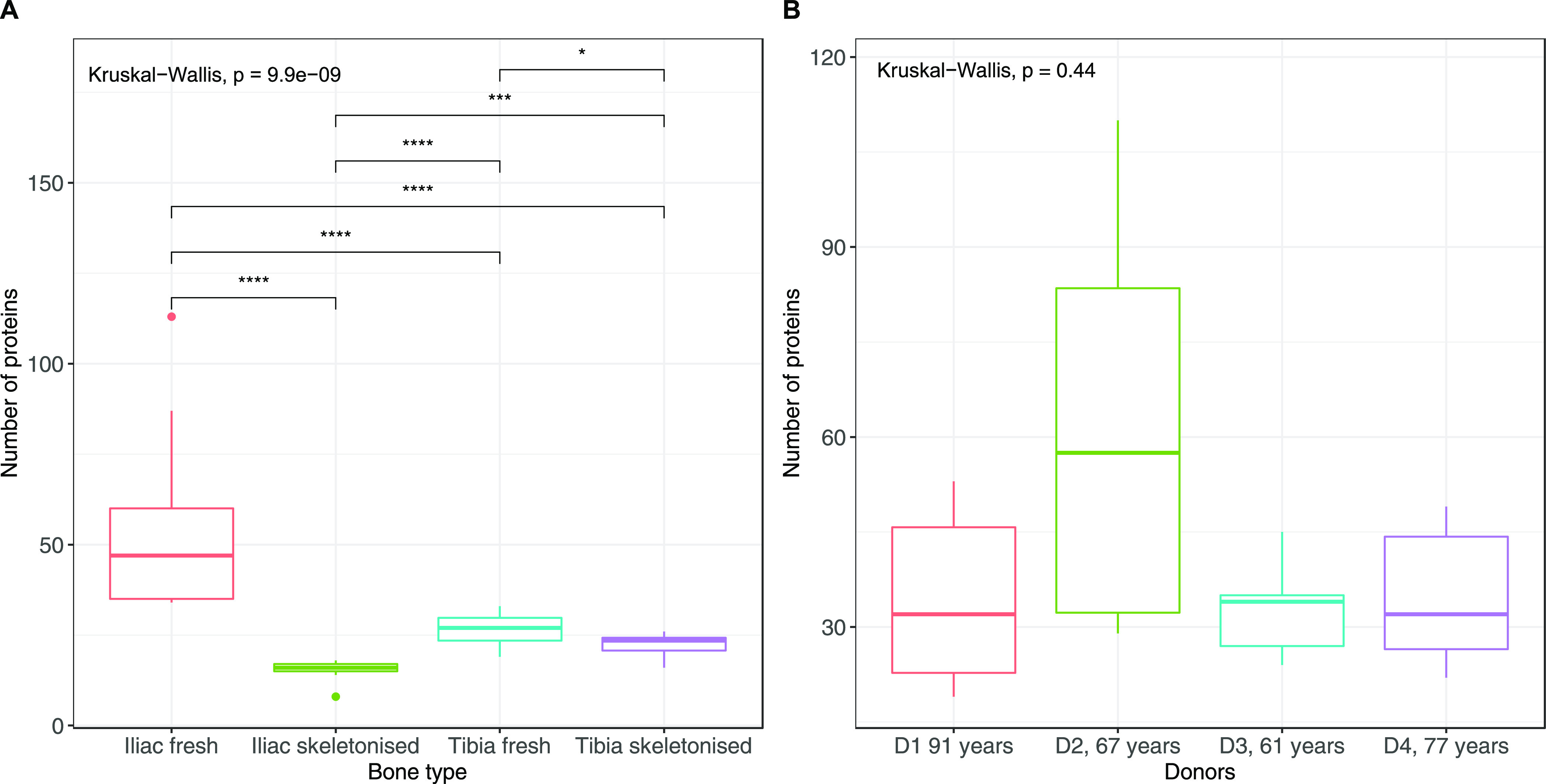
(A) Number of proteins extracted from each sample. Samples were grouped according to the bone type. All bone types were significantly different from each other (post-hoc pairwise Wilcoxon-test with corrections for multiple testing). Outliers are represented as pointed dots in the plot [two outliers identified here, one for iliac fresh (sample NP14, see Supporting Information, Table 2 for details) and one for tibia iliac skeletonized group (sample NP45, see Supporting Information, Table 2 for details)]. (B) Number of proteins extracted from fresh samples. Samples were grouped according to the donor. None of the donors resulted in being significantly different from each other (post-hoc pairwise Wilcoxon-test with corrections for multiple testing, *p* value >0.05).

**Figure 3:**
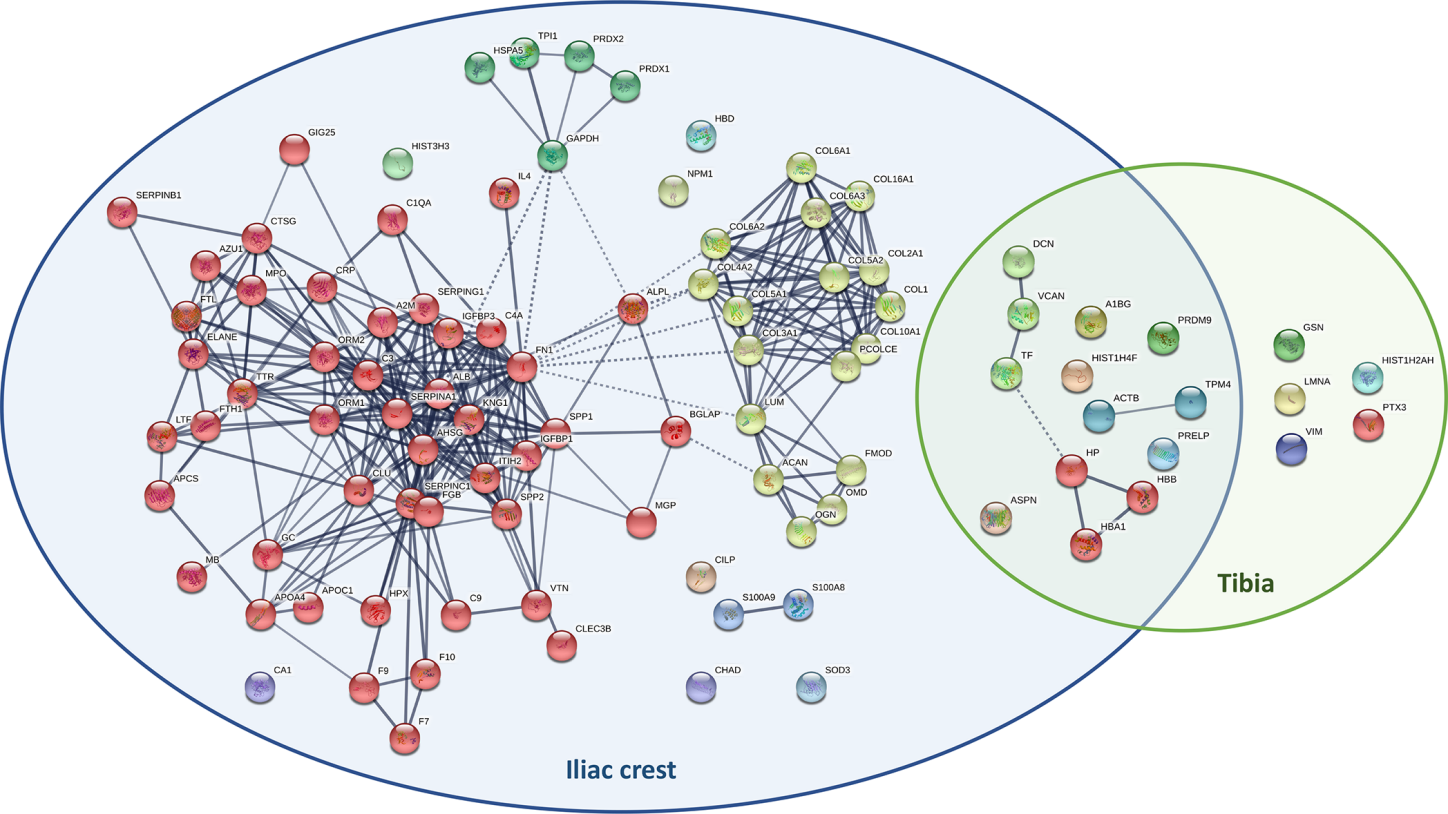
Venn diagram to represent STRING protein networks of proteins significantly more abundant in fresh iliac samples (left) and fresh tibia samples (right) than in their skeletonized counterparts. Proteins shared between the two categories are represented in the middle. Immunoglobulin proteins (gene names IGHA1, IGHG2, IGHG3, IGKC, and IGLC2) were not found with STRING and are not represented in the figure. In the iliac crest category, red cluster represents plasma proteins, yellow cluster represents collagens and bone-related proteins, and green cluster represents ubiquitous proteins. No obvious clusters were identified for the shared proteins and for the ones belonging to the tibia category.

Fresh samples were found to have a significantly greater protein diversity than skeletonized samples (Figure 2A), and, in particular, fresh iliac samples were the richest samples analyzed, both in terms of proteome diversity (average of 55 distinct proteins in iliac fresh samples *vs* 27 for tibia fresh, 15 for iliac skeletonized, and 23 for tibia skeletonized, see Supporting Information, Data 3 for details) and protein relative abundances (Supporting Information, Data 4). Of note, fresh iliac samples were characterized by the presence of 73 proteins found exclusively in that sample type and not in tibia samples (Supporting Information, Data 3). Among these, 38 are blood/serum proteins, and the remainder are bone-specific/mineral-binding (17), extracellular matrix (1), ubiquitous (13), and muscle (4) proteins. When considering protein-relative abundances, among the 116 proteins with significantly different relative abundances between the various bones and sampling times (*i.e.*, fresh *vs* skeletonized), 105 (90.5%) were more abundant in the fresh iliac samples, eight (6.9%) in the skeletonized tibia samples, two (1.7%) in the fresh tibia samples, and one (0.9%) in the skeletonized iliac samples (Supporting Information, Data 4). When comparing iliac fresh and skeletonized samples, 96 proteins including all protein types [bone (29), muscle (2), ubiquitous (12), cartilaginous (1), extracellular matrix (2), and plasma (50) proteins] showed significantly different abundances in the two groups; in all cases, these were more abundant in fresh than in skeletonized samples (Figure 3 and Supporting Information, Data 5). Comparison of the fresh and skeletonized tibia samples revealed 23 proteins with significantly different expression in the two groups, of which 19 were more abundant in the fresh samples [bone (5), muscle (3), ubiquitous (3), and plasma (8)] and four in the skeletonized samples [bone (1), cartilaginous (1), extracellular matrix (1), and plasma (1)] (Figure 3 and Supporting Information, Data 5).

Comparison of inter-individual proteome variability of fresh bones only showed that samples collected from D2 had a richer proteome variety (average number 62 for D2 *vs* 34, 33, and 35 for D1, D3, and D4, respectively), although this difference was not statistically significant (Figure 2B). Within D2, the iliac sample had a notably richer proteome than the tibia sample. In particular, we found 37 proteins uniquely expressed in D2 fresh iliac crest and nowhere else, and for those proteins, we found enriched KEGG pathways for complement and coagulation cascades (Benjamini *p* = 5.5 × 10^–6^), Biocarta pathways for the classical component pathway (Benjamini *p* = 1.6 × 10^–2^), and GO term biological processes for platelet degranulation (Benjamini *p* = 3.1 × 10^–6^), negative regulation of endopeptidase activity (Benjamini *p* = 5.5 × 10^–6^), fibrinolysis (Benjamini *p* = 2.2 × 10^–4^) and complement activation, classical pathway (Benjamini *p* = 5.7 × 10^–3^). Looking at inter-individual protein abundance variability (Supporting Information, Data 6), we found 41 proteins with differences in the relative abundance among the donors. Of these, 36 (87.8%) were more abundant in D2 and were, respectively, bone (6), muscle (3), ubiquitous (8), extracellular matrix (1) and plasma (18) proteins, two each in D1 (both plasma proteins) and D4 (both bone proteins) (4.9%) and one in D3 (cartilaginous protein) (2.4%).

### 3.3 Influence of Environment on Bone Proteome

**Figure 4:**
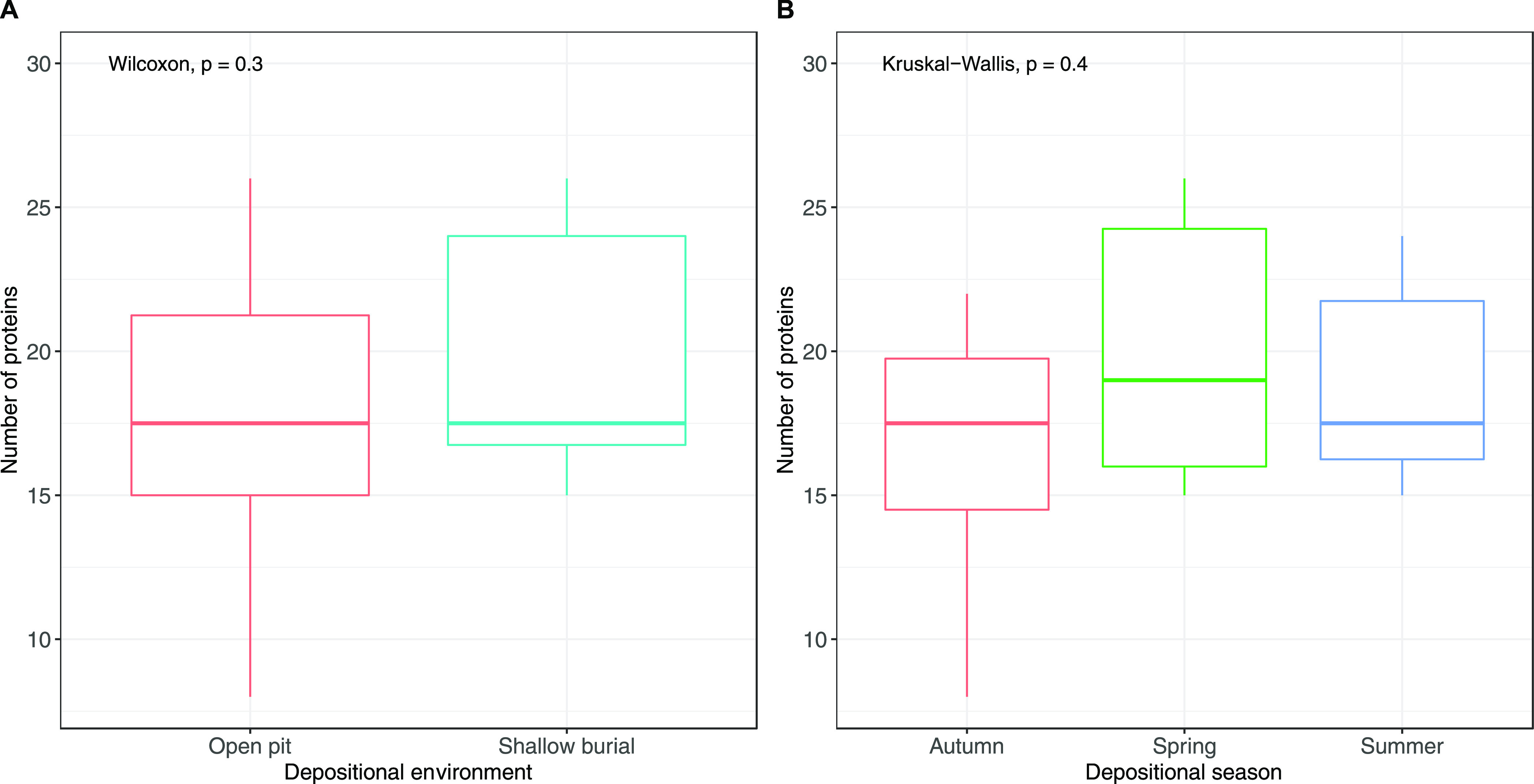
Number of proteins extracted from skeletonized samples, grouped by (A) depositional environment or (B) placement season. No significant differences were detected (Wilcoxon and Kruskal–Wallis *p* value >0.05).

Comparison of samples from different depositional environments (open pits *vs* shallow burials) showed no significant differences in the number of extracted proteins (*p* = 0.3; Figure 4A). Comparison of the relative protein abundances in these two groups revealed only four proteins with a different mean abundance for the two environments (three proteins were more abundant in shallow burials, one protein was more abundant in open pit placements, Supporting Information, Data 7). A test for association between the number of recovered proteins and the season of placement found no significant differences (*p* = 0.4; Figure 4B).

### 3.4 Potential Proteomic Biomarkers for Human PMI Estimation

**Figure 5:**
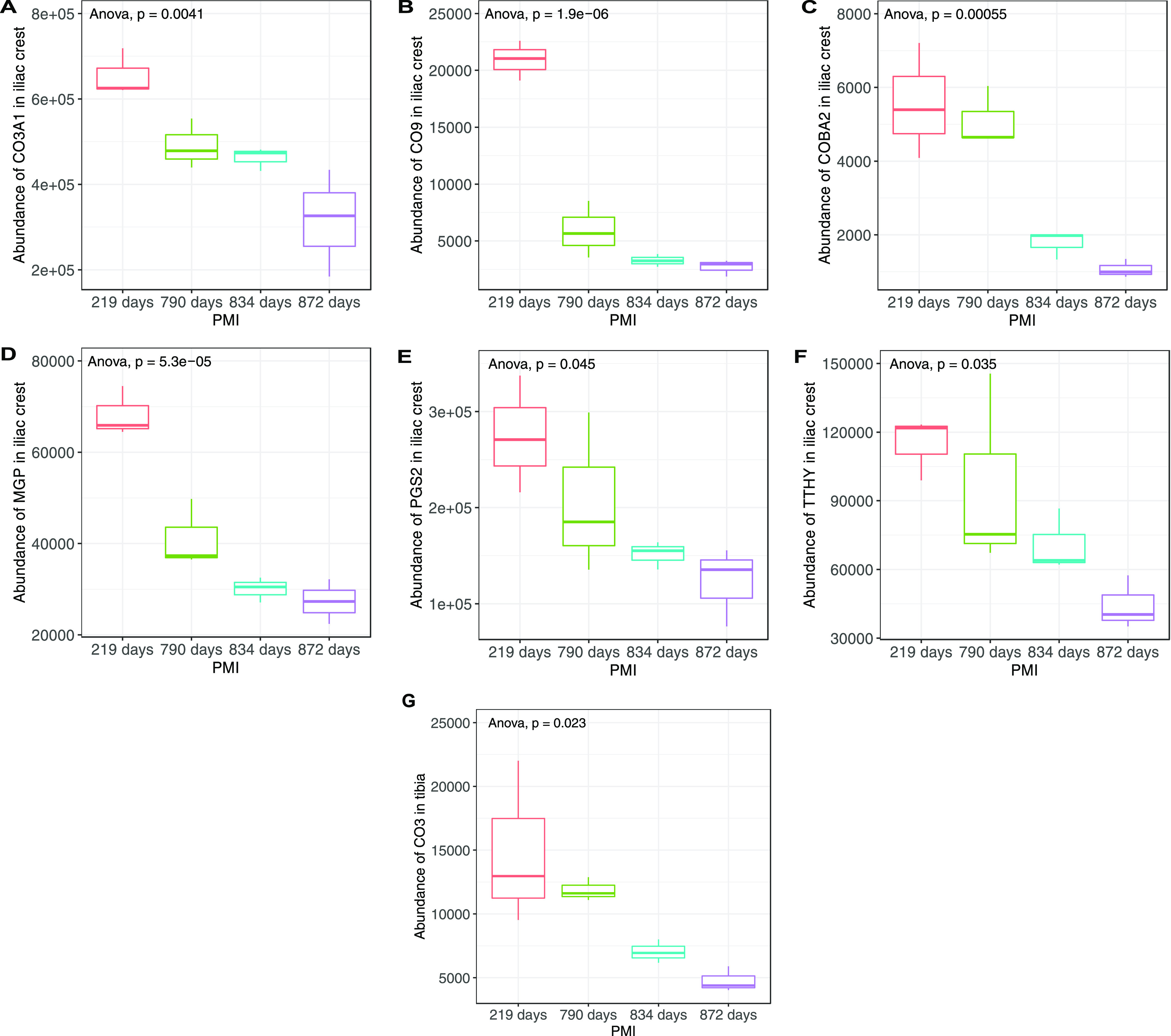
Abundance of (A) CO3A1, (B) CO9, (C) COBA2, (D) MGP, (E) PGS2, and (F) TTHY protein in iliac crest-skeletonized samples and of (G) CO3 in tibia-skeletonized samples with increasing PMIs. Groups are significantly different from each other (ANOVA *p* value <0.05).

No association was found between the number of extracted proteins and the PMI of the samples. However, significant decreases in the abundance of collagen alpha-1(III) chain (CO3A1; *p* = 0.0041), complement C9 (CO9; *p* = 1.9 × 10^–6^), collagen alpha-2(XI) chain (COBA2; 0.00055), matrix Gla protein (MGP; *p* = 5.3 × 10^–5^), decorin (PGS2; *p* = 0.045), and transthyretin (TTHY; *p* = 0.035) in iliac crest (Figure 5A–F) and of complement C3 (CO3) in tibia (*p* = 0.0.023; Figure 5G) were observed when comparing the protein abundances of the four skeletonized samples.

### 3.5 Potential Proteomic Biomarkers for Human AAD Estimation

**Figure 6:**
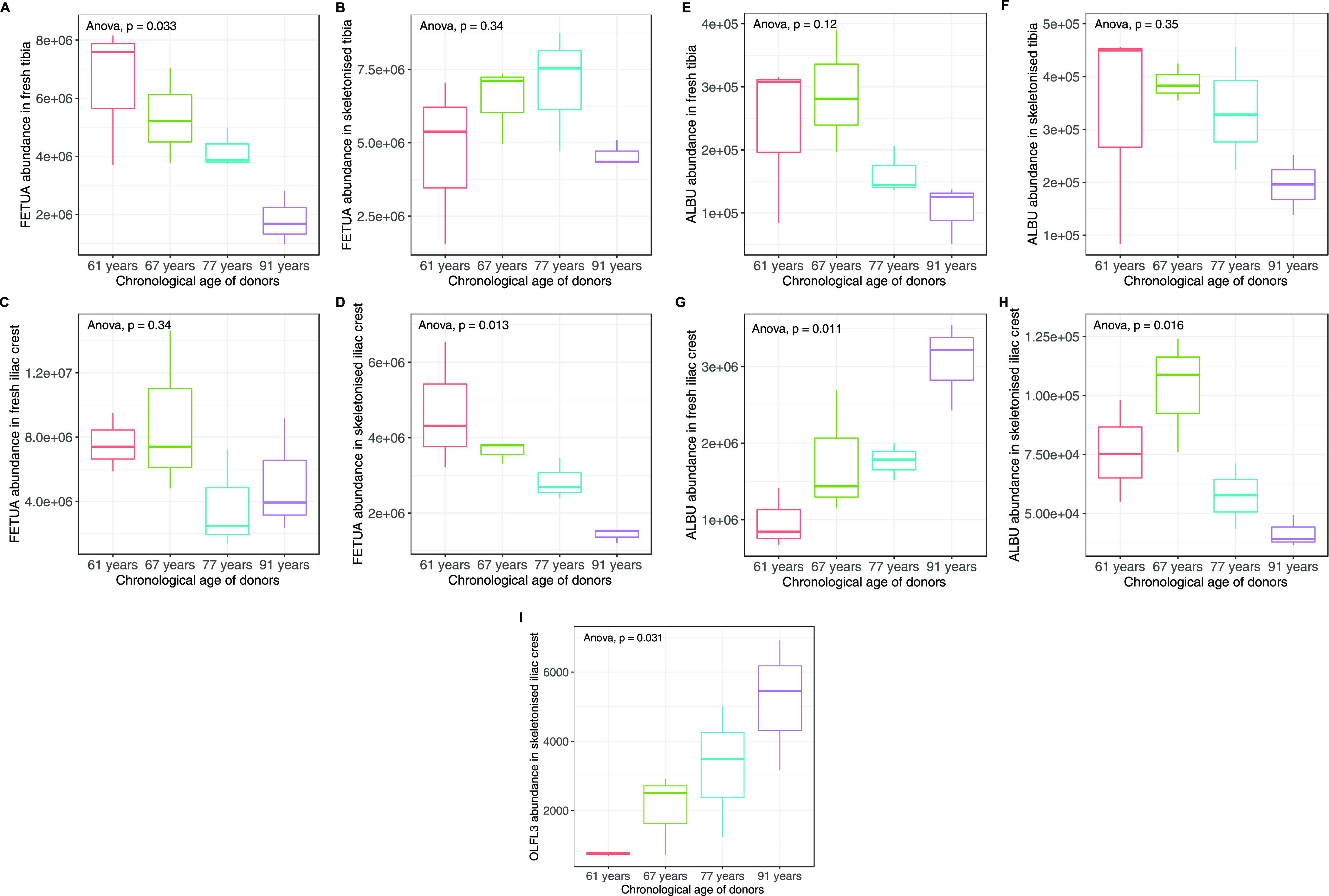
Relative abundance of fetuin-A in (A) fresh tibia, (B) skeletonized tibia, (C) fresh iliac crest, and (D) skeletonized iliac crest samples, of albumin in (E) fresh tibia, (F) skeletonized tibia, (G) fresh iliac crest, and (H) skeletonized iliac crest samples and of (I) olfactomedin like-3 in skeletonized iliac crest samples, arranged by the chronological age of the donors. ANOVA *p* value was reported for each plot. Only (A,D,G,H,I) resulted in being statistically significant.

The relative abundance of fetuin-A was found to be negatively associated with AAD in fresh tibia (*p* = 0.033) and in skeletonized iliac samples (*p* = 0.013). Skeletonized tibia samples showed lower levels for the oldest donor and higher levels for the others, but this result was not statistically supported (*p* = 0.34). Iliac fresh samples showed similar levels in D2 and D3 and lower values for D1 and D4 (*p* = 0.34; Figure 6A–D).

Significant differences in albumin abundance were found between different donors for both fresh (*p* = 0.011) and skeletonized (*p* = 0.016) iliac samples (Figure 6E–H). In particular, fresh iliac samples showed a positive association with AAD, while fresh and skeletonized tibia samples both showed a negative relationship with AAD, although these results were not significant [Figure 6; (*p* = 0.12 and 0.35, respectively)].

Additionally, a significant increase in the abundance of olfactomedin-like protein 3 (OLFL3) was observed in skeletonized iliac samples with increasing AAD (Figure 6G; *p* = 0.031).

### 3.6 Micro Computed Tomography (μCT)

μCT was conducted on skeletonized samples with the aim to evaluate the potential relationship between structural parameters and surviving proteins. The limited sample size did not permit statistical comparison between samples, however sample D2 showed considerably higher values for BV/TV and BMD. For instance, these values are 0.97 for tibia and 0.96 for iliac crest in D2, while the mean values for the remaining samples are 0.92 and 0.93 for tibia and iliac crest, respectively. This results in increased vBMD. We cannot exclude that this variation could be related to the increased protein variety and abundance observed in this individual. Measured values can be found in Supporting Information (Table 3).

## 4 Discussion

The availability of bone samples both before and after decomposition from the same body donors is currently very limited. To the best of our knowledge, the present study is the only one conducted to date that has included such samples from controlled decomposition experiments, allowing us to examine the effects of taphonomic processes such as diagenesis and bioerosion on the bone protein profiles. Due to the limited number of donors, in this study, we chose to analyze multiple extractions from the same bones, which increases the number of data points to model in small samples. However, when treating each extraction from the same body donor as an independent observation, it is important to consider potential confounding by pseudo-replication. Pseudo-replication has been extensively discussed in several fields,^42−45^ and consequently, in this study, it is necessary to apply caution in the interpretation of the observed results. The present work highlights the presence of certain protein biomarkers from a small number of donors, which could be useful for future research on the relation between PMI and protein profiles, and reveals how the mineral matrix and processes of bioerosion likely play a crucial role in the survival of proteins during decomposition. As such, this study represents an important proof of concept for the analysis of the effects of both biological and taphonomic processes on the human bone proteome.

In this study, we identified specific proteins that significantly decreased with increasing PMI: complement C3 for tibia and collagen alpha-1(III) chain, complement C9, collagen alpha-2(XI) chain, matrix Gla protein, decorin, and transthyretin for iliac crest. Four of the identified proteins are classified as bone structural/functional proteins (CO3A1, COBA2, MGP, and PGS2), and three are plasma proteins (CO3, CO9, and TTHY). Previous work, conducted on animal proxies (pigs) left to decompose for a maximum of 6 months,^32^ revealed a similar trend of consistently decreasing protein abundances over time but for different proteins: hemoglobins, transferrins, triosephosphate isomerase, collagen alpha-2(V) chain, and albumin. Both studies showed a reduction in the abundances of plasma and ubiquitous proteins, but reduction in bone structural/functional proteins was observed only in the current study. It is known that certain mineral-binding proteins (including structural ones) are susceptible to taphonomic processes of decay and diagenesis with prolonged PMIs.^46^ The difference in duration and in the depositional environment and local climate between the pig study and the present study, and the resulting longer exposure to taphonomic processes, could therefore explain the different trends in mineral-binding protein abundances reduction that we observed. Analysis of human femoral bones from a cemetery context by Prieto-Bonete and colleagues^34^ also revealed a distinct reduction in the amount of structural and functional proteins in the highest PMI samples (13–20 years). Among the list of proteins identified in their study as biomarkers for prolonged PMIs, COBA2 was the only one that was also found in our study, showing a similar inverse association with increasing PMIs. Overall, these findings suggest that COBA2 could be a good candidate for PMI estimation of human skeletonized remains, due to its durability over time and under different taphonomical conditions.

Our study found that the abundance of OLFL3, an osteoblast secreted extracellular matrix glycoprotein,^47^ was positively associated with AAD in skeletonized iliac samples, adding a previously unreported protein to the list of potential biomarkers for AAD. Previous studies on animal and archaeological human bones identified a negative correlation between serum fetuin-A and AAD^21,48^ and proposed fetuin-A as a potential biomarker for AAD.^31^ The present study identified a similar negative relationship in fresh tibia and skeletonized iliac crest samples but not in fresh iliac and skeletonized tibia. In addition to fetuin-A, several studies showed that serum albumin concentration is negatively correlated with AAD.^49,50^ The present study found a non-significant negative correlation both in fresh and skeletonized tibia samples but an opposite and significant trend in fresh iliac samples. Considering the relatively small sample size, it is difficult to interpret these findings. Our results therefore represent a proof of concept for further research involving a larger sample size that might clarify whether fetuin-A and albumin are consistently negatively correlated with AAD in humans in different bone types and therefore could be used as a biomarker for AAD.

BMD is known to vary between different parts of the skeleton,^51^ and iliac bone is generally less densely mineralized than tibia. In our skeletonized samples, the iliac crest showed lower BMD than tibia in three out of four individuals. D3 was the only donor that showed a slightly higher BMD for iliac crest than for tibia. The highly vascularized (*e.g.*, supplied with blood vessels) and less densely mineralized fresh iliac samples yielded greater variety and abundance of proteins, particularly of those expressed specifically in plasma. The proteomes recovered from skeletonized iliac samples demonstrated that significant protein decay occurred in this bone. The denser and less porotic fresh tibia samples yielded lower protein variety and abundances by comparison to the fresh iliac samples. Comparison of the fresh tibia samples with the skeletonized tibia samples showed that protein decay also occurred in this bone but not to the degree observed in the iliac crest. These results suggest that natural differences in BMD and blood perfusion between the iliac crest and the midshaft anterior tibia influence both the *in vivo* protein variety and abundance as well as the preservation of the bone proteome throughout the taphonomic processes of decomposition. A combination between higher vascularization of the iliac crest and lower BMD exposed this bone to significant deterioration as a result of taphonomic processes over time, resulting in the limited inter-individual differences observed in the skeletonized iliac samples. Protein extraction from the dense anterior midshaft tibia indicated less taphonomic deterioration over time with mineral-binding and bone-associated proteins less prone to deterioration in skeletonized tibia in comparison with skeletonized iliac crest. While taphonomic processes of decomposition are known to affect BMD in humans, and can differentially affect skeletal elements,^52,53^ our results suggest that higher initial (natural) BMD may have a protective effect on proteins within the mineral matrix. BMD could theoretically affect the variety and abundance of specific non-collagenous proteins that can either bind to the calcium ions or the collagen in the mineral matrix,^26^ thereby affecting the overall protein profile.

The potentially protective environment of the bone mineral matrix for specific proteins may be related to the effects of the decomposer community and physicochemical environment on the decomposition of human remains. A less dense matrix would facilitate leaching while promoting the movement of decomposer microbes throughout the bone. Microbial-induced bioerosion, which is characterized by the chemical dissolution of mineral components of bone followed by the microbial enzymatic attack of organic components of bone, is thought to be one of the main causes of bone diagenesis.^54^ The movement of decomposer microbes might be restricted to the external surfaces of more densely mineralized bone. The effects of the decomposer microbial community may be further influenced by the location of the bones. The position of the iliac crest in the trunk of the body exposes this bone to the moisture and a large gastrointestinal microbial community of the gut, which is known to translocate during decomposition.^55,56^ The iliac crest, therefore, is located in a microhabitat that is more favorable for microbial decomposition. In contrast, the tibia is located further from the trunk in limbs that are more prone to desiccation during decomposition. The body position during decomposition of the four donors was flexed and allowed the anterior tibiae to remain elevated above decomposition fluids excreted from the trunk. Desiccation of the soft tissues around the anterior tibiae was observed early on during decomposition of both open pit placements. Desiccated, densely mineralized bone is unlikely to be favorable for microbial decomposition.

Inter-individual comparisons revealed that fresh tibia samples from all four donors had greater inter-individual reproducibility than fresh iliac samples, whereas fresh iliac samples among different individuals were less reproducible, being characterized by an increased abundance and variety of serum proteins as previously reported. However, this phenomenon was exacerbated particularly in D2, the donor showing the highest BMD measurements for both anatomical areas. The enrichment pathways observed for the proteins uniquely present in D2 fresh iliac crest showed processes of inflammation and coagulation that could be related with the carcinogenic history of the donor and with potential metastasis originating in close proximity (*i.e.*, iliac crest) to the carcinoid tumor. Moreover, the presence of specific proteins such as granins suggests a neuroendocrine origin of the tumor, which appears to be associated with the carcinoid tumor that this donor had. The available medical information on D2 suggests that certain conditions and treatments received in the years prior to death could have been associated with changes in BMD, including chemotherapy treatment for cancer, prolonged consumption of calcium lactate,^57^ and possible use of probiotics as adjuvant during cancer treatment.^58,59^ While no conclusive interpretation can be drawn regarding the relationship between increased BMD and medical history for this specific cohort, these results indicate that it is important to consider that medical treatments could induce physicochemical and structural modifications of bone matrix that could affect PMI estimation based on proteomics. Similarly, this concept can be extended to metabolic disorders that significantly affect bone matrix and that are exacerbated with increasing age such as osteoporosis.^60^

The greater BMD of D2 may have allowed for a stronger *in vivo* embedding of mineral-binding and bone-related proteins within the mineral matrix, resulting in the greater proteomic variety of this class of proteins, in addition to the previously discussed blood proteins, particularly in D2 fresh iliac samples. This effect of BMD on protein linkage in bones may be explained in a similar way to what is normally observed between organic matter content and soil density (which is often a function of clay content), where a positive relationship exists between the two variables. In fact, clay particles tend to carry a negative charge to bind with nutrient cations such as calcium and potassium, and these bonds can protect proteins from decomposition and even from extreme environmental conditions such as autoclaving.^61^

Comparison of samples from open pit placements with samples from burials, as well as comparison of season of placement in this study, found no significant differences. While analyses of archaeological remains have revealed differences in protein recovery related to depositional environment,^23,26,33^ it is possible that due to the relatively short duration of this experiment, such environmental effects were not measurable in this study. It is also possible that the two depositional environments did not produce distinct enough conditions (Supporting Information, Table 1) to cause noticeable differences in the preservation of the biomolecules.

The preliminary indications from this study support previous findings that specific proteins decay at different rates, strengthening the potential for developing bone proteomic PMI estimation methods. COBA2 appears to be a good candidate for PMI estimation of skeletonized remains, together with CO3A1, PGS2, and MGP. The blood proteins CO3, CO9, and TTHY may be good candidates for shorter PMI estimation (*i.e.*, before the complete degradation of blood proteins). Our study only partially supported previous studies identifying fetuin-A and albumin as potential biomarkers for AAD estimation, and additionally found OLFL3 being positively correlated with AAD.

At the same time, our findings suggest that taphonomic (*e.g.*, microbial bioerosion) and biological (*e.g.*, variation in BMD and perfusion) variables play a significant role in the survival of proteins. While the sample size is relatively small, the findings point toward potentially significant effects of inter-individual variation associated with health conditions and medical treatment on the variety and abundance of recovered proteins. The results of both inter-individual and intra-skeletal comparisons in our study also suggest that higher BMD may promote attachment of a greater abundance and variety of mineral-binding proteins. Intra-skeletal differences in BMD appear to lead to distinct differences in the variety and abundance of preserved (and extracted) proteins. The attachment of proteins within a more densely mineralized bone matrix may protect them during microbial bioerosion and diagenesis. Based on these indications, we recommend including standard measurement of BMD and targeting a combination of different biomarkers (*i.e.*, abundances of selected plasma proteins and bone-specific proteins) in future work. Overall, our results emphasize the limitations of developing methods and models based on animal proxies since farmed animals rarely show the degree of inter-individual dietary activity and disease-related variation that humans do, and BMD and degree of perfusion of bones differ between species.^62^ Moreover, these results emphasize the importance of conducting replication studies in larger human samples, representing a broader range of robust biomarkers for PMIs and AAD, as well as sampling different bones, to better understand how different types of proteins and different parts of the human skeleton are affected by biological variations and taphonomic processes and to build and validate predictive models for PMI and AAD estimation.

Finally, preliminary evaluation of the inter-skeletal differences we observed suggests that for future development of proteomic PMI estimation methods, the iliac crest bone may be a more suitable sampling target for relatively fresh remains of forensic interest and for archaeological studies specifically targeting the serum-proteins, due to the presence of greater protein variety of bone-marrow proteins. Specific burial conditions, such as dry burial environments, anaerobic environments, and certain post-mortem treatments of the body (such as embalming procedures) can limit the amount of bone diagenesis,^63,64^ thereby promoting the survival of bone proteins across archaeological timeframes. In such circumstances, the iliac crest may provide better results than the tibia to detect pathologies and infections associated with the bone marrow. The midshaft tibia may be a more suitable sampling target for skeletonized remains or those in a state of advanced decomposition, due to the better survival of collagen and mineral-related proteins that could be ultimately used for developing new biomolecular methods for PMI/AAD estimation for forensic purposes.

## Supporting Information Available

Environmental characteristics at FARF; human body donations with additional information and description of the depositions; weather data over the course of the experiment period; data recorded by two HOBO Micro Station data loggers located on FARF at 30 min intervals; ADD data during collection of the bone samples and samples taken for proteomics; μCT data collected after decomposition, including vTMD, material volume (BV)/total volume (TV), and vBMD values (PDF)
133 quantifiable proteins extracted from Progenesis and their localization and binding site according to Uniprot (XLSX)
Top 20 enriched GO terms for the biological process, molecular function, and cellular component of the proteins extracted from all the samples (XLSX)
Proteins identified in the four bone types (iliac fresh and skeletonized, tibia fresh, and skeletonized) (XLSX)
Relative abundances of proteins extracted from all samples only and grouped by the bone type (iliac fresh, tibia fresh, iliac skeletonized, and tibia skeletonized) (XLSX)
Proteins more abundant in iliac fresh samples than in iliac-skeletonized samples and more abundant in tibia fresh samples than in tibia-skeletonized samples (XLSX)
Relative abundances of proteins extracted from fresh samples only and grouped by donor (D1, D2, D3, and D4) (XLSX)
Relative abundances of proteins extracted from skeletonized samples only and grouped by deposition type (open pit *vs* shallow burial) (XLSX)

## Notes

The mass spectrometry proteomic data have been deposited to the ProteomeXchange Consortium *via* the PRIDE^65^ partner repository with the data set identifier PXD019693 and 10.6019/PXD019693.
