# Supplementary material for: Human Bone Proteomes before and after Decomposition: Investigating the Effects of Biological Variation and Taphonomic Alteration on Bone Protein Profiles and the Implications for Forensic Proteomics
Source: J Proteome Res. 2021 Mar 8;20(5):2533–46. doi: 10.1021/acs.jproteome.0c00992 (PMC8155572; doi:10.1021/acs.jproteome.0c00992)
Supplement: Supplementary file 1 — pr0c00992_si_001.pdf [file pr0c00992_si_001.pdf]

SUPPORTING INFORMATION

The Human Bone Proteome Before and After Decomposition: Implications for Forensic Research

Hayley L. Mickleburgh, Ed Schwalbe, Andrea Bonicelli, Haruka Mizukami, Federica Sellitto, Sefora Starace, Daniel J. Wescott, David O. Carter, Noemi Procopio

[Contents]

Supporting Materials and Methods: Including environmental characteristics at Forensic Anthropology Research Facility (FARF), human body donations with additional information, and description of the depositions.

Supporting Table 1: Weather data over the course of the experiment period. Data were recorded by two HOBO Micro Station data loggers located on FARF at 30-minute intervals.

Supporting Table 2: ADD data during collection of the bone samples and samples taken for proteomics.

Supporting Table 3: µCT data taken before burials including volumetric tissue mineral density (vTMD), material volume (BV)/total volume (TV) and volumetric bone mineral density values (vBMD).

Supporting Data 1: List of 133 Quantifiable proteins extracted from Progenesis and their localisation and binding site according to Uniprot.

Supporting Data 2: Top 20 enriched GO terms for Biological Process (first sheet), Molecular Function (second sheet) and Cellular Component (third sheet) of the proteins extracted from all the samples.

Supporting Data 3: List of proteins identified in the four bone types (iliac fresh and skeletonized, tibia fresh and skeletonized).

Supporting Data 4: Relative abundances of proteins extracted from all samples only and grouped by bone type (iliac fresh, tibia fresh, iliac skeletonized, tibia skeletonized).

Supporting Data 5: List of proteins more abundant in iliac fresh samples than in iliac skeletonized samples (first sheet) and more abundant in tibia fresh samples than in tibia skeletonized samples (second sheet).

Supporting Data 6: Relative abundances of proteins extracted from fresh samples only and grouped by donor (D1, D2, D3, D4).

Supporting Data 7: Relative abundances of proteins extracted from skeletonized samples only and grouped by deposition type (open pit vs. shallow burial).

Supporting Materials and Methods

Environmental characteristics at Forensic Anthropology Research Facility (FARF)

FARF's terrain consists of shallow clay-rich soil overlying deposits of limestone with grassland punctuated by woodlands of primarily oak and cedar trees[1]. The region has a semi-humid climate with hot summers and moderate winters. The mean average temperature is 19.4°C (68°F), but temperatures of 32°C (90°F) or greater occur in every month but December and January. Freezing is uncommon, occurring on average 36 days per year, but low temperatures reaching the freezing mark have been reported in all but five months (May through September)[2]. Annual precipitation is 81 cm, but the region experiences frequent droughts and flash flooding. The soils in the area are primarily shallow, stony and overlay Cretaceous limestone. The body donations used in this study were placed in an area of FARF with Rumple-Comfort Association, Ungulating (RUD) type soil, which is relatively shallow and rocky, with clay top soils. This soil is relatively alkaline with high carbonate content and relatively low organic matter. Because of the high clay and rock content, the soil at FARF has relatively low permeability to air and water movement[3–5].

Human body donations

Donation 1

Donation 1 is the willed body donation of a 91 year old female. Donation 1 was placed on 29 April 2015 to decompose unclothed in an open pit throughout the duration of the experiment. The skeletonized remains were retrieved on 3 December 2015. A metal cage was placed over the body to protect it from large scavengers.

Bone samples were collected on 28 April 2015 (B1A) and 3 December 2015 (B1C). The mid-shaft tibia was observed to be hard, dense and of ivory white colour during drilling of the B1A sample. The iliac crest was likewise observed to be hard, dense and of ivory white colour during drilling of the B1A sample. Both mid-shaft tibia and iliac crest B1C samples displayed discoloration of the bone surface consistent with contact with soil, and were both observed to be hard and dense during drilling.

Donation 2

Donation 2 is the willed body donation of a 67 year old female with an extensive documented history of breast cancer and metastases from carcinoid tumours of unknown location. Mastectomy of both breasts was performed at 16 and 13 years prior to death. Bowel resection was performed due to a carcinoid tumour 15 years prior to death. Metastases from carcinoid tumours occurred thereafter until death. Chemotherapy treatment was given between 2 years and 7 months prior to death. Further medical information indicates prolonged consumption of calcium lactate supplements (daily for 15 years prior to death), and possible regular consumption of probiotics throughout adulthood. Bilateral nephrostomy tubes and ureter stents were placed due to blockage associated with carcinoid tumour growth and metastases. During excavation, an ellipsoidal calcified mass measuring approximately 5 cm max. length was recovered from the lower abdominal area (likely a bladder stone). Dietary

information indicates a healthy diet overall, and general avoidance of processed foods and milk during adulthood.

Donation 2 was buried unclothed in a small oval pit. Bone samples were collected and the body was buried on 7 May 2015 (B2A). The skeletonized remains were excavated on 16 and 17 August 2017 and bone samples were again collected (B2C).

The mid-shaft tibia was observed to be *very* hard, dense and of ivory white colour during drilling of the B2A sample. The iliac crest was observed to be hard, dense and of ivory white colour during drilling of the B2A sample. Both mid-shaft tibia and iliac crest B2C samples displayed some discoloration of the bone surface consistent with contact with soil, and were both observed to be very hard and dense during drilling.

<u>Donation 3</u>

Donation 3 is the willed body donation of a 61 year old female who underwent a hysterectomy approximately 33 years prior to death. Donation 3 was buried unclothed in a small oval pit. Bone samples were collected and the body was placed on 24 June 2015 (B3A). The skeletonized remains were excavated on 21 and 22 August 2017 and bone samples were again collected (B3C).

The mid-shaft tibia and iliac crest were observed to be of lower density than the other donations, and of cream colour during drilling of the B3A samples. Both mid-shaft tibia and iliac crest B3C samples displayed discoloration of the bone surface consistent with contact with soil, and were both observed to be of lower density than the other donations during drilling.

<u>Donation 4</u>

Donation 4 is the willed body donation of a 77 year old female. This body donation was placed to decompose unclothed in an open pit throughout the duration of the experiment. A metal cage was placed over the body to protect it from large scavengers. Bone samples were collected on 19 October 2015 (B4A) and the body was placed on 20 October 2015. The skeletonized remains were retrieved on 9 March 2018 and bone samples were again collected (B4C).

The mid-shaft tibia was observed to be hard, dense and of ivory white colour during drilling of the B4A sample. The iliac crest was likewise observed to be hard, dense and of ivory white colour during drilling of the B4A sample. Both mid-shaft tibia and iliac crest B4C samples displayed discoloration of the bone surface consistent with contact with soil, and were both observed to be hard and dense during drilling.

Description of the depositions

The burials in this study were very shallow, meaning that temperature differences between open pit placements and burials are likely to have been minimal. All donors were placed in an area with the same soil type. The two burials were immediately covered with soil, while both open pit placements accumulated soil and debris at the bottom of the pits over time, which

led to the covering of the pelvic sampling area with a small amount of soil (the tibia sampling area remained exposed).

Supporting Table 1. Weather data over the course of the experiment period. Data were recorded by two HOBO Micro Station data loggers located on FARF at 30-minute intervals.

Year	Month	Total precipitation (cm)	Average low (Celsius)	Average high (Celsius)
2015	April (only 29 and 30 April)	0	8.89	25.00
	May	34.14	19.56	27.93
	June	6.55	20.91	31.46
	July	1.63	22.29	34.44
	August	1.6	22.03	35.81
	September	9.55	20.17	33.67
	October	19.91	15.07	29.46
	November	3.68	10.61	21.33
	December	5.82	5.95	20.00
2016	January	2.64	2.81	16.81
	February	2.9	13.84	21.63
	March	9.68	11.43	24.09
	April	16.46	14.17	26.07
	May	38.48	18.05	28.03
	June	8.38	22.22	33.33
	July	14.35	24.25	36.16
	August	21.11	23.32	33.28
	September	8.05	21.35	32.35
	October	0.69	16.02	30.09
	November	6.17	11.78	24.37
	December	7.9	7.67	17.76
2017	January	11.18	6.20	20.05
	February	6.76	10.99	24.4
	March	8.46	13.80	25.38
	April	2.92	15.22	27.83
	May	7.54	17.69	30.81
	June	6.22	22.85	34.22
	July	2.69	23.82	37.17
	August	32.99	23.53	34.53
	September	9.68	19.61	31.96
	October	4.19	13.57	27.20
	November	2.92	11.31	24.09
	December	9.27	4.21	16.38
2018	January	0.64	1.02	15.48
	February	2.72	8.27	17.72
	March (only 1-9 March)	0.25	10.19	22.90

Supporting Table 2. ADD data during collection of the bone samples and samples taken for proteomics.

**Samples**	**Samples for proteomics**	**Date of collection**	**Total ADD (accumulated degree days)**	**Stage of Decay**[9]
B1A-tibia	D1_TF_A<br>D1_TF_B<br>D1_TF_C	28-04-2015	0	Fresh
B1A-iliac	D1_IF_A<br>D1_IF_B<br>D1_IF_C	28-04-2015	0	Fresh
B1C-tibia	D1_TS_A<br>D1_TS_B<br>D1_TS_C	03-12-2015	5225.50	Skeletonized
B1C-iliac	D1_IS_A<br>D1_IS_B<br>D1_IS_C	03-12-2015	5225.50	Skeletonized
B2A-tibia	D2_TF_A<br>D2_TF_B<br>D2_TF_C	07-05-2015	0	Fresh
B2A-iliac	D2_IF_A<br>D2_IF_B<br>D2_IF_C	07-05-2015	0	Fresh
B2C-tibia	D2_TS_A<br>D2_TS_B<br>D2_TS_C	17-08-2017	17828.40	Skeletonized
B2C-iliac	D2_IS_A<br>D2_IS_B<br>D2_IS_C	17-08-2017	17828.40	Skeletonized
B3A-tibia	D3_TF_A<br>D3_TF_B<br>D3_TF_C	24-06-2015	0	Fresh
B3A-iliac	D3_IF_A<br>D3_IF_B<br>D3_IF_C	24-06-2015	0	Fresh
B3C-tibia	D3_TS_A<br>D3_TS_B<br>D3_TS_C	22-08-2017	16817.06	Skeletonized
B3C-iliac	D3_IS_A<br>D3_IS_B<br>D3_IS_C	22-08-2017	16817.06	Skeletonized
B4A-tibia	D4_TF_A<br>D4_TF_B<br>D4_TF_C	19-10-2015	0	Fresh
B4A-iliac	D4_IF_A<br>D4_IF_B<br>D4_IF_C	19-10-2015	0	Fresh
B4C-tibia	D4_TS_A<br>D4_TS_B<br>D4_TS_C	09-03-2018	16848.23	Skeletonized
B4C-iliac	D4_IS_A<br>D4_IS_B<br>D4_IS_C	09-03-2018	16848.23	Skeletonized

Supporting Table 3: µCT data taken before burials including volumetric tissue mineral density (vTMD), material volume (BV)/total volume (TV) and volumetric bone mineral density values (vBMD).

Donor	Bone	Age	$_v$TMD (gHAcm^{-3})	BV/TV	$_v$BMD (gcm^{-3})
1	Tibia	91	1.744	0.910	1.587
1	Iliac crest	91	1.715	0.923	1.583
2	Tibia	67	1.787	0.974	1.740
2	Iliac crest	67	1.780	0.962	1.713
3	Tibia	61	1.775	0.938	1.665
3	Iliac crest	61	1.769	0.950	1.681
4	Tibia	77	1.781	0.914	1.628
4	Iliac crest	77	1.707	0.928	1.584

Supporting References

1. Barnes, P. W. *et al.* Soils, topography and vegetation of the Freeman Ranch. *Free. Ranch Publ. Ser.* **1**, 29 (2000).
2. Dixon, R. Climatology of the Freeman Ranch, Hays County, Texas. *Free. Ranch Publ. Ser.* **3**, 1–9 (2000).
3. Carson, D. Soils of the freeman ranch, Hays County, Texas. *Free. Ranch Publ. Ser* **4**, 1–11 (2000).
4. Fancher, J. P. Evaluation of Soil Chemistry in Human Decomposition Sites. (Texas State University, 2015).
5. Gillham, D., Young, P. & Long, M. Atlas of freeman ranch. (Texas State University, 2003).
6. Keller, A., Nesvizhskii, A. I., Kolker, E. & Aebersold, R. Empirical statistical model to estimate the accuracy of peptide identifications made by MS/MS and database search. *Anal. Chem.* **74**, 5383–5392 (2002).
7. Nesvizhskii, A. I., Keller, A., Kolker, E. & Aebersold, R. A statistical model for identifying proteins by tandem mass spectrometry. *Anal. Chem.* **75**, 4646–4658 (2003).
8. Szklarczyk, D. *et al.* STRING v11: protein–protein association networks with increased coverage, supporting functional discovery in genome-wide experimental datasets. *Nucleic Acids Res.* **47**, D607–D613 (2019).
9. Megyesi, M. S., Nawrocki, S. P. & Haskell, N. H. Using accumulated degree-days to estimate the postmortem interval from decomposed human remains. *J. Forensic Sci.* **50**, 1–9 (2005).
